# Supplementary material for: Knowledge, attitude, and practice of vasoactive agents infusions: Development and psychometric properties of a questionnaire with chinese clinical nurses
Source: PLoS One. 2025 May 28;20(5):e0312068. doi: 10.1371/journal.pone.0312068 (PMC12118911; doi:10.1371/journal.pone.0312068)
Supplement: S1 File — (DOCX) [file pone.0312068.s001.docx]

**S1 File**:

**Summary of Items Deleted During the Item Screening Process**

| **Items** | **R Value** | **Factor Loading** |
| --- | --- | --- |
| K13. Are you aware that central venous access is preferred for vasoactive agent infusion, with peripheral large vein access as an option in emergencies? | 0.215 |  |
| K9. Are you aware of the key aspects of patient condition that should be closely monitored during vasoactive agent use? | 0.123 |  |
| P29. When adjusting the dose of vasoactive agents, I accurately and thoroughly record the drug name, dosage, concentration, infusion rate, change time, blood pressure, heart rate, and rhythm | 0.225 |  |
| K10. Are you aware that an infusion pump should be used for administering vasoactive agents? |  | <0.40 |
| K18. Are you aware of the pH of commonly used vasoactive agents |  | <0.40 |
| K19. Are you aware of the half-life of commonly used vasoactive agents? |  | <0.40 |
| K20. Are you aware of the onset time of commonly used vasoactive agents? |  | <0.40 |
| K21. Are you aware of the metabolic pathways of commonly used vasoactive agents? |  | <0.40 |
| K22. Are you aware of the initial dose for commonly used vasoactive agents? |  | <0.40 |
| K23. Are you aware of the routine adjustment doses for commonly used vasoactive agents? |  | <0.40 |
