# Supplementary material for: Knowledge, attitude, and practice of vasoactive agents infusions: Development and psychometric properties of a questionnaire with chinese clinical nurses
Source: PLoS One. 2025 May 28;20(5):e0312068. doi: 10.1371/journal.pone.0312068 (PMC12118911; doi:10.1371/journal.pone.0312068)
Supplement: Attachment — Submitted filename: Response to Reviewers.docx [file pone.0312068.s004.docx]

**Response to reviewer comments**

| **Reviewer comments** | **Response to reviewer comments** |
| --- | --- |
| **Journal requirements** | |
| 1. Please ensure that your manuscript meets PLOS ONE's style requirements, including those for file naming. | Thank you for your helpful comments. We will ensure that the manuscript meets PLOS ONE's style requirements, including those for file naming. Regarding the financial disclosure, we confirm the following:  “Supported by Sichuan Science and Technology Program (2022YFS0632).”  The funders had no role in study design, data collection and analysis, decision to publish, or preparation of the manuscript.  We will include this amended Role of Funder statement in the cover letter as requested, and you can update the online submission form accordingly.  Thank you for your guidance. |
| 2. Thank you for stating the following financial disclosure:  “Supported by Sichuan Science and Technology Program(2022YFS0632).”  Please state what role the funders took in the study.  If the funders had no role, please state: "The funders had no role in study design, data collection and analysis, decision to publish, or preparation of the manuscript."  If this statement is not correct you must amend it as needed.  Please include this amended Role of Funder statement in your cover letter; we will change the online submission form on your behalf. |  |
| **Reviewer #1:** | |
| 1. The authors conducted a rigorous procedure to develop a valid and reliable instrument (KAP-AIQ) to measure the knowledge, attitude and practices related to vasoactive infussion among nurses in mainland china. the data from pilot and main study article were congruent and support the conclusion of this study. Generally, KAP-AIQ has the potential for wide application in clinical nursing research | Thank you very much for your positive and constructive feedback. We are pleased to hear that you found the methodology and findings of our study to be rigorous and that the KAP-AIQ instrument has potential for wide application in clinical nursing research. Your comments are greatly appreciated, and we are grateful for your thoughtful evaluation of our work. |
| 2.Data analysis was appropriate. |  |
| 3 the authors lprovide underlying data suppoorting their findings. |  |
| 4. The manuscript is presented using simple English and easy to read and understand. |  |
| **Reviewer #2:** | |
| **INTRODUCTION SECTION**  **Comment 1:** Consider rephrasing to "Vasoactive agents are medications that regulate blood vessel tone and blood flow." **Comment 2:** "By targeting vascular smooth muscle and endothelial cells, they modulate..." **Comment 3:** "Between 10% and 54% of ICU patients receive vasoactive agents for conditions such as sepsis, heart failure, and organ failure." **Comment 4:** "Moreover, the dose and type of vasoactive drug are associated with patient mortality." **Comment 5:** "Nurses are responsible for all aspects of vasoactive medication management, including..." **Comment 6:** "Due to the complexity of vasoactive agents, nurses face challenges in making timely clinical decisions when administering these medications." **Comment 7:** "A recent observational study found that Australian nurses often maintain MAP above target levels, potentially leading to adverse effects from high-dose vasoactive agents." **Comment 8:** "This poses a significant risk to patient safety, especially for those with hemodynamic instability." | **Respond comment 1:** Thank you for the suggestion. We agree that this phrasing provides greater clarity. We have revised the sentence as follows: "Vasoactive agents are medications that regulate blood vessel tone and blood flow."  **Respond comment 2:** We appreciate the reviewer’s feedback. This phrasing enhances the specificity of the explanation. We have updated the sentence to: "By targeting vascular smooth muscle and endothelial cells, they modulate vasoconstriction or vasodilation, influencing blood pressure and organ perfusion." |
| **Paragraph 2:** Consider combining comments 1 and 2 to improve flow. | **Respond (combined with comments 1 and 2):**  We have updated the sentence to:  Vasoactive agents are medications that regulate blood vessel tone and blood flow. By targeting vascular smooth muscle and endothelial cells, they modulate vasoconstriction or vasodilation, influencing blood pressure and organ perfusion[1]. Between 10% and 54% of patients admitted to intensive care units (ICUs) have been reported to receive vasoactive agents for conditions such as sepsis, heart failure, and organ failure. Moreover, factors such as the dose and type of vasoactive drug used are associated with patient mortality. For example, Septic shock patients with a <−50% vasoactive-inotropic score (VIS) response had significantly higher ICU mortality (HR 2.07, 95% CI 1.61–2.66, p < 0.001) compared to those with a ≥50% response, especially after receiving vasoactive-inotropic therapy for more than 24 hours[2-4]. |
| **Paragraph 3:** The transition from the Australian study to the Chinese context could be smoother. Perhaps add a comment like, "While challenges exist globally, the Chinese healthcare system is also facing similar issues." | **Respond :**  We have updated the **Paragraph 3-4** to:  Nurses are entrusted with all aspects of medication management, including the preparation, initiation, administration, titration, weaning, and documentation of vasoactive agents. Given the complexity of these medications, nurses face substantial challenges in making dynamic clinical decisions when administering vasoactive agents to patients[5]. A recent observational study in Australia found that nurses often maintain patients' Mean Arterial Pressure (MAP) above the recommended target range of 60 mmHg to >75 mmHg to prevent hypotension. However, this approach frequently overlooks the potential adverse effects of high doses of vasoactive agents[6]. Similarly, studies in China have demonstrated the feasibility and safety of peripheral infusion of vasoactive agents[7,8]], with the Chinese Nursing Association establishing clinical guidelines for their management[9]. Despite this, adherence to these standards remains inconsistent. Integrating evidence-based guidelines into clinical practice remains a significant challenge. As summarized in a systematic review[10], nurses working in intensive care units globally are responsible for managing vasoactive medications. However, practices vary widely, particularly in areas such as dosing adjustments and patient monitoring, which can increase the risk of medication errors and adverse events in critically ill patients, a particularly vulnerable population. |
| **Paragraph 4:** The comment about the Chinese Nursing Association's standard could be rephrased to be more concise. | **Respond:** Thank you for your suggestion to make the sentence more concise. We have rephrased the comment about the Chinese Nursing Association's standard to improve clarity and brevity. The revised sentence is as follows:  “The Chinese Nursing Association has established a standard for the 'Nursing of Vasoactive Agent Infusion' providing clinical guidelines. However, adherence to this standard in practice remains inconsistent.” |
| **Comment 4:** Consider adding a citation for the mortality association. | **Respond:** We added the appropriate reference for the relationship between vasoactive-inotropic therapy response and mortality. As the following citation:  [4]Jentzer JC, Patel PC, Van Diepen S, Morrow DA, Barsness GW, Kashani KB. CHANGES IN VASOACTIVE DRUG REQUIREMENTS AND MORTALITY IN CARDIAC INTENSIVE CARE UNIT PATIENTS. Shock. 2023;59(6):864-870.doi: 10.1097/SHK.0000000000002123 |
| **Comment 7:** Specify the target MAP levels. | **Respond:** Thank you for your comments. We have now specified the target MAP levels in the manuscript. The revised sentence reads as follows:  “A recent observational study in Australia found that nurses often maintain patients' Mean Arterial Pressure (MAP) above the recommended target range of 60 mmHg to >75 mmHg” |
| Paragraph 4: Explain the specific challenges nurses face in implementing the guidelines | **Respond**: Thank you for your suggestion. We have now specified the specific challenges,and also provided specific references. The revised sentence reads as follows:  There are several challenges when nurses ^,^adhering to guidelines for vasoactive agent administration, including deficiencies in knowledge regarding accurate drug dosage calculation limited recognition of risks such as arrhythmias and organ damage, and difficulties in operating complex equipment like infusion pumps. |
| **Material and Method**  Paragraph 2.1.1: Consider combining the first two sentences to improve flow. | **Respond:** Thank you for your suggestion. Here’s the revised version of the paragraph to improve the flow by combining the first two sentences:  To identify the dimensions of our questionnaire, we applied the Knowledge, Attitude, and Practice (KAP) theory as a guiding framework and subsequently, an expert panel reached a consensus after two rounds of detailed discussions on the conceptualization of these dimensions, informed by a comprehensive literature review and the specific context of clinical nursing care in China. |
| Paragraph 2.1.2: The sentence about the literature review could be simplified. For example, "A literature review was conducted using databases like PubMed, Cochrane Library, Embase, and Scopus to identify relevant studies on vasoactive drug administration." | **Respond：**Thank you for your valuable suggestion. We have simplified the sentence regarding the literature review as per your recommendation. The revised paragraph is as follows:  **Revised Paragraph:**  A literature review was conducted using databases such as PubMed, Cochrane Library, Embase, and Scopus to identify relevant studies on the administration of vasoactive drugs. The search included key terms such as “vasoactive drugs” “vasopressors”“infusion care” “intravenous infusion” “medication administration” “safety management”et al,. The search covered the period from the inception of each database to December 2023. After an initial screening by two independent researchers, the final selection of literature was made through consensus, which included two guidelines, two expert consensuses, one evidence summary, the Health of the People’s Republic of China industry standards (WS/T 433-2023), and the Chinese Nursing Association Group Standard (T/CNAS 22-2021). These sources formed the basis for developing a preliminary questionnaire with 43 items across three dimensions: knowledge, attitude, and practice. |
| Paragraph 2.1.3: The explanation of the correlation coefficient analysis and factor analysis could be simplified. Consider using a table to summarize the key criteria for item selection. | **Respond**：Thank you for your valuable suggestions. We have simplified the description of the item selection process and created an attached table listing the deleted items along with the reasons for their removal for your reference.  **Revised Paragraph:**  In this study, 239 valid questionnaires were used for factor analysis to screen items, with correlation coefficient analysis and factor analysis applied. Items were retained if their correlation with the total scale score (r > 0.4) was statistically significant. Factor analysis was conducted to extract factor loadings, with items deleted if their factor loading was ≤ 0.5 or communality ≤ 0.2. Internal consistency was assessed using Cronbach’s α, and items that reduced α when deleted were removed. Subsequently,in the Knowledge and Behavior dimension, four items were removed based on their low correlation with the total score. After factor analysis, several items with factor loadings below 0.4 were also deleted (See attachment table 1). The remaining 33 items had correlation coefficients ranging from 0.557 to 0.904 with the total questionnaire score, all exceeding 0.4 and showing statistical significance (P < 0.001). |
| Paragraph 2.1.3: The specific items that were excluded could be listed in a table for better readability. | **Respond**：Thank you for the expert's suggestion. The specific deleted items are listed in the appendix table." |
| Paragraph 2.1.5: Consider rephrasing the sentence about the expert evaluation to: "Eleven experts, including critical care specialists, nursing experts, and clinical pharmacists, evaluated the content validity, linguistic clarity, and clinical relevance of the questionnaire items." | **Respond**：Thank you for your helpful suggestion. We have revised the sentence as recommended: "Eleven experts, including critical care specialists, nursing experts, and clinical pharmacists, evaluated the content validity, linguistic clarity, and clinical relevance of the questionnaire items." We believe this revision enhances clarity and improves the flow of the paragraph. |
| Paragraph 2.2.1: The sentence about the sample size could be simplified. For example, "A sample size of at least 330 to 660 participants was estimated to be sufficient for factor analysis." | **Respond**：Thank you for your constructive suggestion. We have simplified the sentence as recommended: “A sample size of at least 330 to 660 participants was estimated to be sufficient for factor analysis.” |
| Paragraph 2.2.2: The sentence about the online recruitment could be rephrased to: "Online recruitment advertisements and questionnaire links were disseminated through WeChat and WenJuanXing to invite voluntary participation from nurses at selected hospitals." | **Respond**：Thank you for your constructive suggestion. We have simplified the sentence as recommended: “Online recruitment advertisements and questionnaire links were disseminated through WeChat and WenJuanXing to invite voluntary participation from nurses at selected hospitals”. |
| Paragraph 2.2.3: The explanation of Cronbach's alpha and ICC could be simplified. For example, "Cronbach's alpha was used to assess the internal consistency of the questionnaire. A value greater than 0.7 indicates good internal consistency." | **Respond**：Thank you for your helpful suggestion. We have simplified the explanation of Cronbach’s alpha and the intraclass correlation coefficient (ICC) as recommended. The revised version is as follows:  Cronbach's alpha was used to assess the internal consistency of the questionnaire. A value greater than 0.7 indicates good internal consistency. The test-retest reliability was assessed using the intraclass correlation coefficient (ICC), which ranges from 0 to 1. A coefficient below 0.4 indicates poor reliability, while a value above 0.75 indicates good reliability. |
| **Statistical Analysis**  The statistical results are well-presented, but for a broader audience, briefly explain the significance of these indices (e.g., Cronbach's alpha, EFA, and CFA). | **Respond:**Thank you for your helpful suggestion. We have revised the paragraph to briefly explain the significance of the statistical indices used in our analysis, making it more accessible for a broader audience. The revised paragraph is as follows:  Exploratory factor analysis (EFA) and confirmatory factor analysis (CFA) were used to examine the construct validity of the questionnaire. EFA helps identify the underlying structure of the data by grouping related items, while CFA is used to confirm whether the data fit the hypothesized structure. |
| **Results**  Paragraph 3.1: Consider rephrasing the sentence about the Likert scale to: "The Knowledge dimension used a 5-point Likert scale ranging from 'Not at all familiar' to 'Very familiar.' The Attitudes dimension used a 5-point Likert scale ranging from 'Strongly disagree' to 'Strongly agree.' The Practices dimension used a 5-point Likert scale ranging from 'Never' to 'Always.'" | **Respond:**Thank you for your insightful suggestion. We have revised the sentence about the Likert scale as per your recommendation to improve clarity. The revised paragraph is as follows:  The final version of the KAP-VAIQ consists of three dimensions: Knowledge with 15 items, Attitudes with 5 items, and Practices with 13 items. The questionnaire can be found in the S1 File (KAP-VAIQ). The Knowledge dimension used a 5-point Likert scale ranging from 'Not at all familiar' to 'Very familiar.' The Attitudes dimension used a 5-point Likert scale ranging from 'Strongly disagree' to 'Strongly agree.' The Practices dimension used a 5-point Likert scale ranging from 'Never' to 'Always.' Scores are assigned from 1 to 5 for each item, with the total questionnaire score ranging from 33 to 165 points. A higher score indicates a greater level of knowledge, attitudes, and practices related to vasoactive agent infusion nursing among the nurses. |
| Paragraph 3.2: Consider rephrasing the sentence about the content validity to: "The questionnaire demonstrated excellent content validity, with an item-level Content Validity Index (I-CVI) ranging from 0.91 to 1.00 and a scale-level Content Validity Index/average (S-CVI/Ave) of 0.98." | **Respond:**Thank you for your insightful suggestion. We have revised the sentence as per your recommendation to improve clarity.The revised paragraph is as follows:  The questionnaire demonstrated excellent content validity, with an item-level Content Validity Index (I-CVI) ranging from 0.91 to 1.00 and a scale-level Content Validity Index/average (S-CVI/Ave) of 0.98. |
| Paragraph 3.3: Consider breaking up the long sentence about the participants' characteristics into shorter sentences. | **Respond:**Thank you for your insightful suggestion. We have revised the paragraph to break up the long sentence and present the participants' characteristics in a clearer and more concise format. The revised paragraph is as follows:  A total of 535 nurses participated in the psychometric assessment, with 478 valid questionnaires collected, yielding a response rate of 89.34%. The majority of participants were women (83.24%). The mean age of the participants was 32.86 years (SD = 7.18), with a median age of 32 years. A significant portion (81.63%) held a bachelor’s degree or higher. All participants were registered nurses, including 425 (88.91%) from tertiary hospitals and 53 (11.08%) from secondary hospitals. Among them, 52 (10.87%) were internal medicine nurses, 142 (29.70%) were surgical nurses, 189 (39.53%) were critical care nurses, 30 (6.27%) were operating room/anesthesia nurses, 21 (4.39%) were pediatric nurses, and 16 (3.34%) were from other specialties. Regarding professional titles, 253 (52.92%) held junior titles, 193 (40.37%) held intermediate titles, and 32 (6.69%) held senior titles or higher. The average clinical nursing experience was 8.32 years (SD = 5.07). |
| Paragraph 3.4: Consider rephrasing the sentence about the Cronbach's alpha to: "The questionnaire demonstrated excellent internal consistency, with a Cronbach's alpha coefficient of 0.96 for the overall scale and 0.96 to 0.98 for the individual dimensions." | **Respond:**Thank you for your valuable suggestion. We have rephrased the sentence about Cronbach's alpha as recommended, improving the clarity and readability. The revised paragraph is as follows:  The questionnaire demonstrated excellent internal consistency, with a Cronbach's alpha coefficient of 0.96 for the overall scale and 0.96 to 0.98 for the individual dimensions. The test-retest reliability was also high, with an intraclass correlation coefficient (ICC) of 0.90 for the overall questionnaire, and ICCs for the individual dimensions ranged from 0.90 to 0.94 (P < 0.05). |
| Paragraph 3.5: Consider rephrasing the sentence about the factor analysis to: "Exploratory factor analysis (EFA) was conducted on 239 samples. The Kaiser-Meyer-Olkin (KMO) measure of sampling adequacy was 0.945, indicating that the data were suitable for factor analysis. Bartlett's test of sphericity was significant (p < 0.001), further supporting the factor analysis | **Respond:**Thank you for your insightful suggestion. We have rephrased the sentence about the factor analysis as recommended.The revised paragraph is as follows:  Exploratory factor analysis (EFA) was conducted on 239 samples. The Kaiser-Meyer-Olkin (KMO) measure of sampling adequacy was 0.945, indicating that the data were suitable for factor analysis. Bartlett's test of sphericity was significant (p < 0.001), further supporting the factor analysis. |
| **Discussion**  Comment 1:You have clearly stated the objective of developing and testing a questionnaire. To strengthen this, emphasize the significance of understanding clinical nurses' knowledge, attitudes, and practices regarding vasoactive agent infusions. For example:  Comment 2:You mention that this study is the first of its kind in this area. Consider elaborating on why this gap existed and how this research contributes uniquely. | **Respond:**Thank you for your suggestion. We have elaborated on why this gap existed and how our study contributes uniquely by addressing the absence of a validated questionnaire for assessing clinical nurses' knowledge, attitudes, and practices regarding vasoactive agent infusions. The revised paragraph emphasizes this gap and the significance of our research.The revised paragraph is as follows:  In this study, we developed and tested a preliminary Chinese questionnaire to assess the knowledge, attitudes, and practices of clinical nurses concerning vasoactive agent infusions. As far as we know, this type of comprehensive, validated questionnaire had never been conducted previously. The gap in the literature stems from the fact that most existing measurements of nurses’ knowledge and practices related to vasoactive agents were based on empirical research and lacked psychometric validation, which affected the accuracy of assessments and limited their external validity and applicability. This gap is critical, as the safe administration of vasoactive agents is contingent upon nurses' precise knowledge and well-founded practices.Our study addresses this gap by offering a reliable and valid instrument, which can inform the design of targeted educational programs and improve clinical practice. By measuring nurses’ knowledge and practices quantitatively, this tool enables the development of more effective educational curricula tailored to the specific needs of clinical nurses, thereby enhancing patient safety and care quality in clinical settings. |
| Comment 3: The link between the questionnaire design and the KAP theory is mentioned but could be elaborated upon. For example, discuss how each component of the theory is operationalized in the questionnaire items. | **Respond:**Thank you for your suggestion to further elaborate on how the components of the KAP theory are operationalized in the questionnaire. We have expanded on this point to clearly explain how each dimension of the theory is represented in the questionnaire items. The revised paragraph is as follows:  According to the KAP theory, knowledge, attitudes, and practices are interrelated, with knowledge forming the foundation for attitude change, which in turn influences the adoption of practices[27].In the context of this study, the Knowledge dimension assesses nurses’ understanding of the physiological mechanisms and safe administration practices for vasoactive agents, while the Attitudes dimension evaluates the nurses’ beliefs and perceptions regarding the importance of proper administration. The Practices dimension measures the actual behaviors exhibited by nurses, including adherence to safety protocols and clinical guidelines. This alignment with the KAP theory makes the questionnaire a robust tool for assessing the cognitive and behavioral components that influence nurses’ practices in vasoactive agent infusion. |
| Comment 4:You discuss the benefits of the questionnaire for administrators and researchers. Strengthen this by providing specific examples of potential initiatives. | **Respond:**Thank you for your suggestion to strengthen the benefits of the questionnaire for administrators and researchers. We have provided more concrete examples of how the questionnaire can be used to implement targeted initiatives.  **Revised sentences:**Based on the assessment results, nursing administrators can implement targeted initiatives to improve nurses' adherence to the standardized management of vasoactive agents. These initiatives may include increasing relevant training, optimizing clinical workflows, enhancing communication among healthcare teams, and ensuring the availability of necessary resources.Additionally, the questionnaire can serve as a tool for evaluating the effectiveness of training programs over time. |
| Comment 5: You’ve identified the limitations well. To improve, suggest practical steps to address these limitations in future research. | **Respond:**Thank you for your valuable feedback.we added some details how to address these limitations as follow:  There are some limitations in this study. Firstly, convenience sampling was used, and the web-based questionnaire management platform indicated that all respondents' IP addresses were located in one province in western China, which may limit the generalizability of the findings. To address this, future studies could consider using a more diverse sampling approach, such as stratified or random sampling, and expanding the geographic scope to include multiple provinces or regions. Secondly, while we assessed content and construct validity, no criterion validity test was applied to this scale. Future research should consider testing the criterion validity of the KAP-VAIQ by comparing it with established instruments or clinical outcomes to further validate its accuracy and relevance. |
| Comment 6: The mention of intelligent technology and organizational safety climate is insightful. Consider expanding this by hypothesizing how these factors might interplay with the KAP dimensions. | **Respond:**Thank you for your suggestion to make our team consideration the link with KAP. We revised as following： Additionally, studies have shown that factors such as intelligent technology and organizational safety climate can enhance nurses’ knowledge through real-time feedback, shape their attitudes toward safety protocols, and improve their practices by providing better resources. |
| Comment 7: In the conclusion, emphasize the broader implications and potential applications of the questionnaire. | **Respond:**Thank you for your suggestion to emphasize the broader implications and potential applications of the questionnaire in the conclusion. We have revised the conclusion to highlight these points.  **Revised Paragraph (Comment 7):**  In conclusion, the KAP-VAIQ has significant implications for improving patient safety and the quality of care in the administration of vasoactive agents. This validated questionnaire can be used to assess and monitor the effectiveness of nursing education and training programs, ensuring that nurses possess the necessary knowledge, attitudes, and practices for safe vasoactive agent infusion. Furthermore, it can be applied in diverse healthcare settings to identify gaps in practice and inform evidence-based interventions. Ultimately, the KAP-VAIQ can play a key role in enhancing the standardization and safety of vasoactive agent administration, contributing to improved patient outcomes. |
| **Reviewer #3:** | |
| **Comments1:**Dear author, I congratulate you on your work. I believe your article will provide a valuable contribution to nursing services. The validity and reliability study of the scale appears to be extremely robust. However, if you could validate the scale's results with the outcomes of other studies, we might better observe the impact of your scale. | **Respond comment 1:** Thank you very much for your kind words and thoughtful feedback. We greatly appreciate your recognition of the validity and reliability of our scale. We agree that validating the results of our scale against the outcomes of other studies would provide further insights into its impact and generalizability.We plan to explore this direction in future research by comparing the results of the KAP-VAIQ with those from similar studies in different critical care nursing unites within China, and even globle.This comparative analysis will help establish the broader applicability of the scale and enhance its validation. We will ensure to report these findings in subsequent publications.Thank you again for your valuable suggestion. We believe that this step will further strengthen the contribution of our work to nursing services. |
